# Failure analysis of primary surgery and therapeutic strategy of revision surgery for complex tibial plateau fractures

**DOI:** 10.1186/s13018-019-1147-4

**Published:** 2019-04-24

**Authors:** Zhe Song, Qian Wang, Teng Ma, Chen Wang, Na Yang, Hanzhong Xue, Zhong Li, Yangjun Zhu, Kun Zhang

**Affiliations:** Department of Orthopaedic Trauma, Hong-Hui Hospital, Xi’an Jiaotong University, No. 76, Nanguo Road, Beilin District, Xi’an City, 710054 Shaanxi Province China

**Keywords:** Tibial plateau fracture, Knee joint, Fracture fixation, Proximal tibia, Revision surgery

## Abstract

**Purpose:**

To analyze the cause of failure of the primary surgery for complex tibial plateau fractures and to define the therapeutic strategy of the revision surgery for the same.

**Methods:**

Twenty-one cases with failure of primary surgery for complex tibial plateau fractures were treated in our hospital from January 2012 to September 2016. There were 13 males and 8 females with an average age of 39.4 years (ranged between 27 and 58 years). Patients presented with different types of complex tibial plateau fractures like Schatzker type V (*n*=9), VI (*n*=12), type 41.C1 (*n*=9), type 41.C2 (*n*=6), and type 41.C3 (*n*=6). The therapeutic strategy for revision surgery in individual patients was decided following careful analysis and accurate assessment of the causes of failure of the primary surgery. All the patients were followed-up with Rasmussen radiographic scores and Hospital for Special Surgery (HSS) knee scores.

**Results:**

All 21 patients underwent clinical and radiological examination after a mean follow-up time of 32.6 months. The average time of fracture healing was 4.5 months (ranged between 3 and 6 months). During the last follow-up, the mean range of motion of knee extension was 2.3° and knee flexion was 123.8°. The mean radiological Rasmussen score was 15.6 points, with an overall success rate of 85.7%. The average HSS knee score was 84.3 points, with an overall success rate of 80.9%.

**Conclusion:**

The common reasons for the failure of primary surgery of complex tibial plateau fractures were inadequate experience of the surgeon, inaccurate diagnosis and management, improper selection of implants, and poor surgical techniques. The key factors to succeed revision surgery were adequate preoperative evaluation, accurate intraoperative procedures, and proper postoperative rehabilitation.

**Level of evidence:**

Level IV, case series, treatment study

## Introduction

Tibial plateau fractures are common intra-articular fractures, accounting for 1–2% of all fractures and up to 8% of all fractures in the elderly population [1, 2]. Fractures that have an impact on the function and stability of tibial plateau usually require surgery. The incidence of complex tibial plateau fractures is increasing worldwide, especially in developed countries, as they occur due to the result of high-energy trauma. The incidence of tibial plateau fractures could be up to 36%, and the complex tibial plateau fractures often occur in young men, leading to different degrees of articular surface compression and displacement along with severe damage of structures around the knee joint [3]. Some postoperative complications, such as instability of knee joint, posttraumatic arthritis, disability of lower extremities, and incapability to work, are usually caused by improper surgical treatment [4].

Nowadays, the operative management of complex tibial plateau fractures is challenging for even the most experienced surgeons, as satisfactory outcomes are not always attainable [5]. The anatomy of the proximal tibia, combined with high-energy trauma, produces complex injury patterns that involve metaphyseal and articular comminution fractures, frequently leading to the loss of integrity of the soft tissue envelope. The degree of comminution of metaphysis and articular surface along with the severity of soft tissue injury reflects the energy transmitted to the bone, leading to unfortunate prognosis [6].

In recent years, the surgical of complex tibial plateau fractures has been greatly improved, while the incidence of complications has been greatly reduced, owing to the advancement in the imaging diagnostic techniques, improvement of surgical techniques, and development of internal fixation devices [7]. However, some patients have failed at the primary surgery and been needed the revision surgery.

The possible reasons for primary surgery failure could be due to the inadequate experience of the orthopedic surgeon, inadequate assessment of these injuries, incomprehensive surgical techniques, and incorrect selection of internal fixation. In view of this situation, we carefully analyzed the clinical data of 21 patients with complex tibial plateau fractures and who completed follow-up. This study aimed to summarize the experience and lessons learned from the failure of primary surgery, to explore the factors that need to be assessed before revision surgery, and to identify effective preventive measures to avoid surgical complications.

## Patients and methods

### Inclusion and exclusion criteria

Inclusion criteria were as follows: adult patients aged 18 years and above with complex tibial plateau fractures and failure of primary surgery, patients with normal neurovascular function before primary surgery, patients with closed fractures, patients with good soft tissue condition, and patients without serious medical diseases and obvious surgical contraindications.

Exclusion criteria were as follows: patients with an open fracture, pathologic fracture, previous medical history of knee fractures and/or injury to knee ligaments, multiple fractures that might affect postoperative rehabilitation, and serious preoperative medical diseases.

### General data

A total of 21 eligible patients were included in this retrospective study conducted from January 2012 to September 2016 (Table 1). This research has been approved by the IRB of the authors’ affiliated institutions. Informed consent was obtained from all the individual participants.

**Table 1:** Main characteristics of the study population

Patient ID	Gender	Age (year)	Type of trauma	Type of fracture (Schatzker classification)	Type of fracture (AO classification)
1	F	39	Fall from height injury	VI	41.C2
2	F	27	Bicycle accident	V	41.C1
3	M	44	Fall from height injury	V	41.C1
4	M	37	Traffic accident	VI	41.C3
5	M	31	Crush injury	V	41.C1
6	F	51	Traffic accident	VI	41.C2
7	M	45	Bicycle accident	V	41.C1
8	F	38	Traffic accident	VI	41.C3
9	M	43	Bicycle accident	V	41.C1
10	M	33	Fall from height injury	VI	41.C2
11	F	34	Crush injury	VI	41.C2
12	F	41	Bicycle accident	VI	41.C3
13	M	43	Traffic accident	V	41.C1
14	M	32	Altercation injury	VI	41.C3
15	F	35	Fall from height injury	V	41.C1
16	M	58	Crush injury	VI	41.C2
17	M	54	Bicycle accident	VI	41.C3
18	F	29	Traffic accident	V	41.C1
19	M	30	Traffic accident	VI	41.C2
20	M	46	Fall from height injury	VI	41.C3
21	M	37	Bicycle accident	V	41.C1

There were 8 female and 13 male patients; 7 patients had fractures on the left side, and 14 had fractures on the right side. The average age of the patients was 39.4 years (range 27–58 years). In this study, the reasons for complex tibial plateau fractures in patients were due to road traffic accidents (six cases), bicycle accidents (six cases), fall from heights (five cases), crush injuries (three cases), and altercation injury (one case). All of the fractures were closed and isolated injuries without any neurovascular injury. We evaluated clinical and radiological outcomes in patients with the Schatzker [8] and the AO [9] classifications. Nine cases presented with Schatzker type V, 12 presented with type VI, 9 presented with type 41.C1, 6 presented with type 41.C2, and 6 presented with type 41.C3 fractures. All the surgeries of these 21 patients were performed by an orthopedic surgeon with more than 20 years of surgical experience.

### Preoperative planning

In this series of patients, the fracture type and failure cause of primary surgery were analyzed and evaluated by radiologic data and surgical information. After that, physical examination, X-ray films and CT scans, or 3D printing models (if necessary) were performed in all the patients before revision surgery. According to these information, the following analyses and evaluations were conducted to further clarify the fracture characteristics and classification before the first surgery, fracture reduction after the first surgery, and the reductive sequence of main bone fragment for the second surgery; to further identify the reduction of articular surface, the presence or absence of the collapse in the articular surfaces, and the presence or absence of compressive fractures in the epiphysis, evaluating the extent of bone defects and the amount of bone graft requirements; to analyze the surgical approach and internal fixation strategy of primary surgery; and to define appropriate surgical approach, the placement of internal fixation, and the fixative direction of main screws during the revision surgery.

### Surgical procedure

Dual incisions of medial and lateral approach were performed for the revision surgery (the original incision should be preserved as far as possible). Primarily, the medial incision (posterior medial approach) was performed to expose the medial implants and the fracture of medial condyle to remove the original hardware, reduce the fracture of the tibial plateau, and fix with dual plates at the anteromedial and posteromedial region. Then, the joint was opened via lateral incision (anterolateral approach) to fully expose the lateral condyle and the lateral articular surface of the tibia and to complete the open reduction of the articular surface and metaphysis after removing the original implants, as well as to carry out internal fixation with anatomical plate of proximal tibia, and bone grafting with artificial bone. The reduction of metaphyseal and diaphyseal fracture, reconstruction of articular surface, and proper positioning of the implants were confirmed approvingly by intraoperative fluoroscopy.

### Postoperative management

Antibiotics and anticoagulants are routinely used intraoperatively and postoperatively. Following surgery, the leg was placed in a foam splint and elevated to relieve the limb swelling. The standard postoperative regimen included the early initiation of motion and muscle strengthening exercises immediately after surgical wound healing. No weight-bearing was allowed until 10–12 weeks after the surgery. The patients were allowed to walk with two crutches without weight-bearing on the affected leg and were encouraged to gradually increase the range of motion using active exercises and a continuous passive motion device with the aim to regain complete extension and 90 degrees of flexion. Full weight-bearing was not allowed until the confirmation of complete fracture healing by postoperative radiography.

**Fig. 1:**
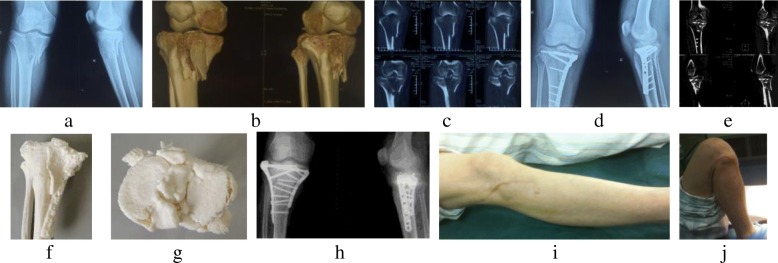
A 54-year-old male patient sustained the tibial plateau fracture caused by bicycle accident (Schatzker V, AO/OTA 41.C1). The first preoperative X-rays in anteroposterior and lateral views (**a**) and CT-scans (**b**, **c**) showed the tibial plateau fractures of medial and lateral condyle and the slight collapse of the lateral articular surface. The second preoperative X-rays in anteroposterior and lateral views (**d**), CT scans (**e**), and 3D printing model (**f**, **g**) showed the widening of the tibial plateau, the collapse of the lateral articular surface, and the poor reduction of the medial plateau. The X-rays in anteroposterior and lateral views (**h**) after the revision surgery indicated the correction of the widening of the tibial plateau, the restoration of lateral articular surface, the satisfied reduction of the medial plateau, and the favorable internal fixation of the anterolateral and posteromedial plate. The functional appearance (**i**, **j**) at 30 months after the revision surgery showed that the nice healing of the wounds and the satisfactory function of THE knee joint

X-ray and/or CT was used at initial presentations as well as at follow-up to assess the degree of maximal joint depression and to check for possible delayed union or nonunion. Radiographic results were assessed using the Rasmussen score, which includes depression, condylar widening, and angulation (varus/valgus) with scores lying between 0 and 18 [10]. Functional recovery was assessed using the knee score of Hospital for Special Surgery (HSS), which includes pain, function, range of motion, muscle strength, flexion deformity, and instability. All complications were also noted. All 21 patients were included in the study and completed the follow-up examination (Fig. 1).

## Results

According to the analysis and summary of the whole clinical and radiographic data of all patients, the causes of failure of primary surgery can be divided into three types and were as follows: faulty diagnosis and treatment (7 patients; 33.3%), inaccurate selection of implants (8 patients; 38.1%), and imperfect surgical techniques (6 patients; 28.6%).

All 21 patients were clinically and radiologically examined after a mean follow-up time of 32.6 months (between 24 and 48 months). The average time of fracture healing was 4.5 months ranging from 3 to 6 months. Of the 21 patients included in the study, 19 patients (90.5%) got the primary wound healing in 2 weeks after the operation. The remaining two patients (9.5%) showed poor wound healing and had superficial infections requiring a regular change of dressing and antibiotic treatment. Late complications were seen in seven patients (33.3%). Among these seven patients, traumatic arthritis with slight pain in knee joint was treated with analgesic drugs in four patients. The remaining three patients showed different degrees of knee dysfunction, where one patient showed improvement after physical therapy and two patients required surgical intervention and eventually regained a normal range of motion. There were no reports of deep infections, limb shortening, delayed or nonunion of fractures, secondary collapse of articular surface, or knee instability and implant breakages.

During the last follow-up, radiological films of all 21 patients showed that the fractures were healed. The mean range of motion of knee extension was 2.3° (measured between − 30 and 10°), and knee flexion was 123.8° (measured between 90 and 135°). The mean radiological Rasmussen score [11] was 15.6 points (ranging between 4 and 18 points) and indicated good clinical outcome. Among these patients, 11 cases had excellent outcome, 7 had good outcome, 2 had fair outcome, and 1 showed poor outcome, with an overall success rate of 85.7%. The average HSS knee scores of 21 patients [12] were 84.3 points (ranging between 53 and 98 points). Ten cases had excellent scores, sevn cases had good scores, three cases had fair scores, and 1 case had a poor score, with an overall rate of 80.9 % (Table 2).

**Table 2:** Follow-up results

Patient ID	Follow-up (month)	Failure cause	Healing time (month)	Extension (degree)	Flexion (degree)	Rasmussen score	HSS score
1	14	Inaccurate selection of implants	4	4	130	18	91
2	15	Inexperience of diagnosis and treatment	3	5	128	18	91
3	18	Inaccurate selection of implants	4	0	122	14	83
4	13	Inexperience of diagnosis and treatment	6	− 30	90	4	53
5	15	Imperfect surgical techniques	4	8	132	18	97
6	16	Inexperience of diagnosis and treatment	4	− 10	100	10	67
7	20	Inaccurate selection of implants	5	5	135	18	95
8	21	Imperfect surgical techniques	4	6	125	16	84
9	18	Inexperience of diagnosis and treatment	5	5	135	18	97
10	15	Imperfect surgical techniques	4	6	126	16	81
11	16	Inaccurate selection of implants	4	7	133	18	92
12	24	Inaccurate selection of implants	6	5	120	14	68
13	20	Inexperience of diagnosis and treatment	4	10	130	18	95
14	18	Inaccurate selection of implants	4	3	126	16	81
15	15	Inexperience of diagnosis and treatment	6	5	130	18	94
16	12	Inexperience of diagnosis and treatment	6	7	135	18	87
17	14	Imperfect surgical techniques	5	− 20	90	8	63
18	16	Inaccurate selection of implants	3	10	135	18	98
19	12	Inexperience of diagnosis and treatment	4	8	130	18	84
20	30	Imperfect surgical techniques	5	5	120	14	79
21	36	Inaccurate selection of implants	4	10	130	16	82

## Discussion

Complex tibial plateau fractures are characterized by severe compression and obvious sinking of both the articular surfaces [1, 2, 10], mainly occurring as a result of high-energy trauma, such as road traffic accidents or falls from height. The high-energy bicondylar tibial plateau fractures are still one of the most difficult fractures to treat and are often complicated by infections, nonunion, compartment syndrome, and osteoarthritis, leading to failure of surgeries [13, 14].

In this study, we defined the tibial plateau fractures of Schatzker type V and VI or AO/OTA type C (C1, C2, and C3) as complex tibial plateau fractures, which was consistent with the viewpoints of most of the scholars [1, 2, 15, 16]. The reasons for this definition are as follows: (1) it is a serious intra-articular fracture, which involves the articular surface, epiphysis end shaft of proximal tibia; (2) it is almost always accompanied by severe soft tissue injury; (3) the anatomical structures around the knee joint are grossly injured, making it difficult to perform the surgery; and (4) there are many postoperative complications even after regular surgical treatment. As a result, complex tibial plateau fractures must be treated through optimal surgery, which requires preoperative planning of surgical procedures by experienced surgeons based on the characteristics and classification of the fractures. Otherwise, it results in some serious postoperative complications and even requires the revision surgery to recover the knee function.

Literature search [17–19] and retrospective analysis of clinical patients in our hospital revealed that the common reasons for the surgical failure of complex tibial plateau fractures were inadequate experience of the surgeon regarding the diagnosis and treatment, inaccurate selection of implants, and imperfect surgical techniques.

Of the 21 patients included in this study, 7 cases (33.3%) required revision surgery because of inadequate experience of the surgeons regarding the diagnosis and surgical treatment of the complex tibial plateau fractures. Some patients were operated only with the assistance of X-rays, obviously causing incomplete evaluation of the fracture and further affecting the planning of the surgery. This in turn led to the failure of the first surgery. Surgeons need to have a correct understanding of the characteristics and types of the fractures as these are the key information for successful surgical management of complex tibial plateau fractures. As a result, we should make the full use of X-rays and CT scans to obtain the stereo image of the fracture and to clarify the complicated fracture lines and multiplanar displacement of the fracture fragments in order to make a more accurate evaluation of the fracture [4, 19].

Eight cases failed because of the inaccurate selection of implants. Surgeons usually could confirm the characteristics of injury and the type of fracture according to radiological imaging (X-ray and/or CT scan) of the patients. However, the wrong choice of internal fixation could give rise to the failure of primary surgery. For example, fixation and fracture reduction would definitely fail in the medial condylar fracture of Schatzker type V fracture of the tibial plateau, if the fracture is only compressed with the anteromedial plate and without the supporting fixation of the posteromedial plate.

Faulty surgical techniques resulted in the failure of primary surgery in six cases. Though the surgeon chooses the appropriate surgical approach and optimal implants during the surgery, it was still hard to achieve the articulation of articular surface, adequate reduction of fractures, reduction of knee dislocation, restoration of limb alignment, and better protection of the surrounding soft tissues due to improper surgical techniques [20]. Consequently, complications such as poor fracture reduction, poor and faulty internal fixation, posttraumatic arthritis, and dysfunction of knee joint occur, leading to the requirement of revision surgery.

Complex tibial plateau fracture is a serious injury of the knee joint and remains very tricky to treat. With the failure of primary surgery, the chances of successful revision surgery also become poor. Therefore, it is necessary to obtain complete imaging data during primary surgery as much as possible. If possible, 3D printing model should be carried out to fully acquire the accurate information of the fractures [21]. Some factors must be considered before planning revision surgery. These factors include not only the injury characteristics and fracture type before the primary surgery and the postoperative reduction of fractures, but also the current condition of fracture healing, the state of implants, local conditions of soft tissues surrounding the knee joint, the function of the knee joint, and the subjective feelings of the patient.

The local conditions of soft tissues and the original surgical approaches around the knee joint should be adequately assessed before selecting the correct surgical approaches for the revision surgery. It is conducive to prevent the incidence of complications of soft tissues by using the original surgical approaches for the revision surgery and to protect the soft tissues intraoperatively as much as possible [22–24]. For the selection of internal fixation, the effectiveness of the original implants should be judged from the results of preoperative analysis and intraoperative findings. The internal implants can be retained if it does not interfere with fracture reduction and effective fixation of implants during revision surgery. However, if the original implants interfere with fracture reduction and internal fixation, they must be removed completely and the new implants should be placed to achieve fracture reduction and internal fixation [25].

Fracture characteristics and injury mechanisms that are considered before primary surgery should also be considered before planning the revision surgery. Furthermore, the revision surgery should be performed to thoroughly remove the callus and scar tissue obstructing the fracture reduction. According to intraoperative findings, optimal reduction of fractures should be done as much as possible. In the meantime, the articular surface was restored through sufficient bone grafting. The broadening of tibia plateau and the varus/valgus of proximal tibia were also corrected through some specific instruments for fracture reduction. We tried our best to solve all the residual problems after primary surgery to ensure recovery of the functions of the knee joints as much as possible.

In this study, we advocated the principle of “early movement, late weight-bearing” for postoperative rehabilitation. The functional exercises of continuous passive motion (CPM ) were allowed as early as possible after surgery, and the active movements of knee joint were initiated under the supervision of surgeons to restore the normal function of the knee joint. The weight-bearing time and strength training of the affected limb were determined by the growth condition of callus, the healing state of bone graft, and the assessment of the bone quality during the follow-up to prevent the premature and excessive stress due to the re-collapse of articular surface, thus re-displacing the fracture [26, 27].

## Conclusion

In summary, what we need to emphasize is that the complex fractures of tibial plateau are not simple injuries. Whether it is the first time to make a diagnosis and treatment strategy, or secondary revision treatment, the surgeon must pay full attention to this type of fracture. The preoperative X-rays and CT images should be evaluated comprehensively to determine the characteristics of the fracture, the degree of comminuted fracture, the direction of the fracture line, and the position and distance of displaced fragments. At the same time, it is very important to assess the ligaments, meniscus, and soft tissues around the knee joint before planning revision surgery. The choice of appropriate surgical approaches and implants, along with the experience of surgeons, plays important roles in the success of revision surgery.

However, our study has limitations of being a retrospective one and has a relatively short follow-up period that needs to be acknowledged.
